# Applicability of granite and quartzite residues in the development of porous adsorbents for effective organic pollutant removal

**DOI:** 10.1007/s11356-026-37913-7

**Published:** 2026-06-10

**Authors:** Pietro Serraglio Figueiredo, Guilherme Luiz Dotto, Edson Luiz Foletto

**Affiliations:** Research Group On Adsorptive and Catalytic Process Engineering (ENGEPAC), Federal University of Santa Maria, 97105–900, Santa Maria, Brazil

**Keywords:** Ornamental rocks, Granite, Quartzite, Alkaline fusion, Adsorption, Thermodynamics, Mechanism

## Abstract

In this work, granite and quartzite residues were converted into amorphous materials through an alkaline fusion process employing KOH as the activating agent. Structural characterization confirmed the significant loss of crystallinity in both precursors, while textural analysis indicated a marked improvement in the pore structure, particularly in the quartzite-derived material. Among the synthesized adsorbents, the amorphized quartzite (AQZ) demonstrated superior adsorption performance compared to the amorphized granite (AGR). The adsorption of crystal violet (CV) onto AQZ was optimized using experimental design, with pH 9 and an adsorbent dosage of 0.9 g L⁻^1^ identified as the most favorable conditions. Kinetic studies showed that the system reached equilibrium within 90 min. Isotherm evaluation revealed a maximum adsorption capacity of 86.24 mg g⁻^1^ at 298 K, confirming the high efficiency of AQZ. Thermodynamic parameters indicated that the adsorption process is spontaneous, exothermic, and thermodynamically favorable, with electrostatic attraction identified as the predominant interaction mechanism. AQZ retained satisfactory adsorption efficiency after four consecutive regeneration cycles. These findings demonstrate that alkaline fusion is a viable strategy for transforming ornamental stone residues into a reusable adsorbent for treating CV-contaminated wastewater.

## Introduction

The ornamental stone sector is essential to the building and architectural industries, yet it generates substantial amounts of solid waste from quarrying, sawing, and surface finishing processes (Bagheri et al. 2024). Within this sector, quartzite is a metamorphic rock largely composed of silica, whose high structural compactness, chemical resistance, and durability under aggressive conditions make its residues attractive for reuse in advanced material applications (Howard 2005). Granite, another extensively processed ornamental stone, is an igneous rock composed mainly of quartz, feldspar, and mica (Momeni et al. 2015). The coexistence of these mineral phases leads to a chemically diverse surface that can contribute to adsorption through various interaction mechanisms (Momeni et al. 2015).

The large-scale extraction and processing of quartzite and granite pose significant environmental challenges, including the generation of fine powders, alteration of natural landscapes, atmospheric particulate emissions, and the potential release of contaminants into soils and aquatic systems (Ahmad et al. 2023). Consequently, the development of sustainable routes for managing these residues has become a priority. The conversion of quartzite and granite wastes into value-added materials, particularly porous adsorbents with low production costs, represents an effective approach to reduce environmental burdens while promoting resource efficiency. Through appropriate physical and chemical modification, these residues can be engineered into porous solids with improved textural properties and accessible active sites, enabling their application in water treatment technologies. Among available treatment methods, adsorption stands out as a robust and straightforward process for removing organic pollutants from aqueous media, owing to its high efficiency and operational simplicity (Netto et al. 2022).

Driven by the growing demand for efficient, sustainable adsorbent materials derived from natural resources, amorphization has emerged as an effective strategy to enhance adsorption performance (Figueiredo et al. 2024). This approach is based on the partial or complete loss of long-range crystalline order, leading to an amorphous structure with increased porosity and a higher density of accessible surface sites, key attributes for adsorption-driven processes. Amorphization is commonly induced by thermal treatment in the presence of solid alkaline agents, which promote structural rearrangement and phase transformation (Demir Delil et al. 2019). Materials modified through this route generally exhibit superior adsorption behavior when compared to their pristine counterparts, particularly for the uptake of emerging contaminants such as pharmaceutical compounds, heavy metal ions, and synthetic dyes (Rossatto et al. 2022).

Among the various classes of water pollutants, synthetic dyes represent a major environmental concern. During industrial dyeing and finishing operations, a significant fraction of these compounds is discharged into aquatic systems without adequate treatment, leading to persistent and widespread water contamination (Vasques et al. 2009). In addition to causing visible discoloration of water bodies, many dyes possess complex molecular structures containing aromatic rings, sulfonated groups, and azo bonds, which are linked to adverse health effects such as toxicity, inflammatory responses, and carcinogenicity (Dilarri et al. 2016; Leite et al. 2020). From a chemical standpoint, dyes can be categorized as anionic or cationic, depending on the electrical charge they assume in aqueous media. This property strongly governs their interaction with adsorbent surfaces (Hassan and Carr 2021).

Anionic dyes, including Congo red (CAS: 573–58–0) and methyl orange (CAS: 547–58–0), typically contain sulfonate (–SO₃⁻) groups that impart a negative charge and high solubility in water. These characteristics often hinder their removal, especially when negatively charged adsorbents are used, due to unfavorable electrostatic interactions (Azam et al. 2022). Conversely, cationic dyes such as methylene blue (CAS: 61–73–4) and crystal violet (CAS: 8004–87–3) bear positively charged functional groups, which favor electrostatic attraction toward negatively charged surfaces, frequently resulting in enhanced adsorption capacities (Corda and Kini 2018). The contrasting ionic nature of these dye classes influences not only adsorption efficiency but also the roles of solution pH, ionic strength, and adsorbent surface chemistry. Therefore, distinguishing between anionic and cationic dyes is a crucial step in the rational design and optimization of adsorbent materials for wastewater treatment.

In this context, the present study investigates the use of quartzite and granite residues as precursor materials for the preparation of porous adsorbents via solid-state alkaline fusion. Initially, the adsorption behavior of the prepared materials was screened using a range of both cationic and anionic dyes to identify the most effective adsorbent–adsorbate systems. Subsequently, the system exhibiting the highest performance was subjected to comprehensive adsorption studies, including kinetic modeling, equilibrium isotherm analysis, and thermodynamic evaluation, aiming to elucidate the mechanisms governing the adsorption process.

## Experimental

### Preparation of amorphous samples

Quartzite (QZ) and granite (GR) residues were obtained from the cutting stage of ornamental stone processing at a local stone fabrication facility located in Santa Maria city (Brazil). Before treatment, the raw materials were mechanically sieved to isolate particles with diameters smaller than 100 µm. The amorphization process was carried out through an alkaline fusion route, in which the powdered precursor materials (QZ or GR) were homogeneously mixed with solid potassium hydroxide (KOH) using a 1:1 mass ratio. The mixtures were then heated to 550 °C and held for 90 min. The resulting amorphized materials were designated as AQZ (amorphized quartzite) and AGR (amorphized granite). Thermal processing was conducted in open crucibles, which were not hermetically sealed, allowing the reaction to proceed under an oxidizing atmosphere. Importantly, KOH was incorporated directly into the dry powdered samples without water addition, ensuring that alkaline activation occurred exclusively under solid-state conditions. After thermal treatment, the samples were allowed to cool naturally to room temperature and subsequently ground in a mortar and pestle to obtain a fine, homogeneous powder. Successive washes with distilled water removed residual alkaline species until the pH reached neutrality. The washed materials were then rinsed with ethanol and dried in a convection oven at 110 °C for 24 h before further characterization and adsorption experiments. All adsorption experiments were conducted on a dry mass basis, and masses were measured with an analytical balance.

### Characterization techniques

The amorphized quartzite (AQZ) and amorphized granite (AGR) samples were subjected to a comprehensive physicochemical characterization. Fourier transform infrared spectroscopy (FTIR) was performed on a Prestige-21 spectrometer (Shimadzu) to identify surface functional groups and chemical bonds in the modified materials. Crystalline phase composition and structural changes induced by the amorphization process were investigated by X-ray diffraction (XRD), using a Miniflex 300 diffractometer (Rigaku), operated with Cu Kα radiation (λ = 1.54051 Å), an accelerating voltage of 30 kV, a current of 10 mA, a step size of 0.03°, and a counting time of 0.5 s per step. Elemental composition of the precursor materials was determined by X-ray fluorescence spectroscopy (XRF) using a Bruker S2 Ranger spectrometer. Textural properties, including specific surface area and pore characteristics, were evaluated from nitrogen adsorption–desorption isotherms obtained by static physisorption measurements using an ASAP 2020 analyzer (Micromeritics). The analyses were performed at − 195.85 °C, and all textural parameters were calculated using the instrument’s proprietary software. For the adsorbent exhibiting the highest adsorption performance, additional studies were conducted to evaluate the influence of solution pH, adsorbent dosage, and the point of zero charge (pHzcp). The pHzcp was determined to identify the pH at which the material’s surface exhibits neutral charge, guiding selecting appropriate operating conditions. This assay was carried out over a pH range of 1–12 using a NaCl electrolyte solution and a fixed adsorbent concentration of 1 g L⁻^1^. The suspensions were agitated at 150 rpm for 24 h in a mechanical shaker, and all experiments were performed in triplicate. Initial pH adjustments were made using a calibrated pH meter and standardized 0.1 mol L⁻^1^ HCl and NaOH solutions. After equilibration, the final pH values were recorded, and the pHzcp was calculated as the arithmetic mean of the plateau region where the pH remained constant.

### Adsorption assays to optimize pH and adsorbent dosage

Initial adsorption trials were carried out using representative cationic and anionic dyes as model contaminants, namely Congo Red (CR), Methyl Orange (MO), Methylene Blue (MB), and Crystal Violet (CV), to evaluate the adsorption behavior of the AQZ and AGR materials. Aqueous dye solutions were prepared with an initial concentration of 50 mg L⁻^1^. The solution pH was adjusted to 4 for the anionic dyes (CR and MO) and to 9 for the cationic dyes (MB and CV), according to their ionic characteristics. The experiments were performed in an orbital shaker at 150 rpm for 2 h. After equilibration, the suspensions were centrifuged at room temperature for 10 min to ensure complete separation of the solid adsorbents from the liquid phase. Based on the results of these preliminary screening tests, the adsorbent–dye combinations with the highest removal performance were selected for subsequent studies. These systems were further examined using a statistical experimental design approach to optimize key operational variables, namely solution pH and adsorbent dosage. The remaining dye concentration in the supernatant was quantified by UV–visible spectrophotometry at 590 nm (Marco-Brown et al. 2018). The adsorption capacity (*q*) (Eq. 1) and the percentage removal (*R*) (Eq. 2) were calculated using the equations presented below.

1 $$q=\frac{\left({C}_{i}-{C}_{f}\right) V}{{m}_{ad}}$$

2 $$R=\frac{\left({C}_{i}-{C}_{f}\right)}{{C}_{i}}100$$

where *Cᵢ* (mg L⁻^1^) represents the initial dye concentration, ${C}_{f}$(mg L⁻^1^) is the final concentration after adsorption, *V* (L) corresponds to the solution volume, and ${m}_{ad}$(g) is the mass of adsorbent used.

A central composite rotatable design (CCRD) was employed to investigate and optimize the adsorption process. The experimental matrix consisted of three replicates at the center point and four axial experiments, considering two operational factors: solution pH and adsorbent dosage. The pH values explored in the design ranged from 4 to 10, while the adsorbent concentration varied between 0.25 and 1.0 g L⁻^1^. The adsorption capacity (*q*) and the dye removal efficiency (*R*) were selected as the response variables for model construction and evaluation. Model adequacy, statistical relevance, and predictive capability were assessed using analysis of variance (ANOVA) and the Fisher F-test. All data processing and statistical analyses were conducted using Statistica® 8.0.

### Parameters estimation for kinetic models

Adsorption kinetic studies were conducted under the optimized conditions defined by the experimental design. All experiments were performed in triplicate, with initial dye concentrations ranging from 50 to 350 mg L⁻^1^. The kinetic behavior of the adsorption process was analyzed by adjusting the experimental data to the pseudo-first-order (Eq. 3), pseudo-second-order (Eq. 4), and Avrami (Eq. 5) models (Wang and Guo 2020). Parameter estimation and data fitting were performed using Origin® 2019.

3 $${q}_{t}={q}_{e}(1-{e}^{-{k}_{1}t})$$

4 $${q}_{t}=\left(\frac{{q}_{e}^{2}{k}_{2}t}{1+{q}_{e}{k}_{2}t}\right)$$

5 $${q}_{t}={q}_{AV}(1-{exp}^{(-{k}_{AV}.{t}^{n}})$$

where, *q*_*t*_, *q*_*e,*_ and *q*_*AV*_ are the adsorbed quantities (mg g^−1^) at time* t* (min), at equilibrium, and at the Avrami model, respectively; *k*_*1*_ is the rate constant of the pseudo-first-order model (min^−1^), *k*_*2*_ is the rate constant of the pseudo-second-order model (g mg^−1^ min^−1^), *k*_*AV*_ (min^−1^) is the Avrami constant;* n* is the fractional exponent.

### Parameters estimation for isotherm models

Adsorption equilibrium studies were performed under the optimal operational conditions established by the experimental design. The experiments were conducted using initial dye concentrations ranging from 0 to 450 mg L⁻^1^ at different temperatures (298, 308, 318, and 328 K). The equilibrium data were analyzed by applying the Freundlich (Eq. 6), Langmuir (Eq. 7), and Temkin (Eq. 8) isotherm models (Girish 2025) to describe the adsorption behavior. The parameters for each model were determined by nonlinear regression using Origin® 2019.

6 $${q}_{e}={K}_{F}{C}_{e}^{1/{n}_{F}}$$

7 $${q}_{e}=\frac{{Q}_{max}{K}_{L }{C}_{e}}{1+{K}_{L }{C}_{e}}$$

8 $${q}_{e}={\beta}_{T} \mathrm{l}\mathrm{n}({K}_{T }{C}_{e})$$

where, *K*_*F*_ is the constant of the Freundlich isotherm (mg g^−1^) (mg L^−1^)^−1/nF^, ${n}_{F}$ is the exponent of Freundlich, ${Q}_{max}$ is the maximum adsorption capacity (mg g^−1^), ${K}_{L}$ is the constant of Langmuir (L mg^−1^), ${\beta}_{T}$ (J mol^−1^) represents the Temkin parameter related to the adsorption energy, and ${K}_{T}$(L mg^−1^) is the Temkin constant.

### Thermodynamic study

Thermodynamic parameters were determined from equilibrium adsorption data obtained at 298, 308, 318, and 328 K. The Van’t Hoff approach was applied to evaluate the standard thermodynamic functions, including entropy (*ΔS⁰*, kJ mol⁻^1^ K⁻^1^), enthalpy (*ΔH⁰*, kJ mol⁻^1^), and Gibbs free energy (*ΔG⁰*, kJ mol⁻^1^), using Eqs. (9), (10), and (11) (Girish 2025).

9 $${K}_{e}=\frac{{K}_{F}\rho }{1000}(\frac{{10}^{6}}{p}{)}^{(1-\frac{1}{nF})}$$

10 $$ln\left(K_e\right)=-\frac{\Delta H^\circ}{RT}+\frac{\Delta S^\circ}R$$

11 $$\triangle G^\circ=\triangle H^\circ-T\triangle S^\circ$$

### Regeneration and reuse assays

The recyclability and regeneration efficiency of the adsorbent were assessed through multiple adsorption–desorption cycles conducted until saturation was reached. Desorption was performed using absolute ethanol as the eluent, with successive washes until no residual color was observed in the solution. The regenerated material was then thoroughly rinsed with distilled water to remove any remaining ethanol. All regeneration experiments were carried out under the optimized conditions defined by the experimental design, and the adsorbent performance was monitored over four consecutive reuse cycles. The possibility of leaching of alkali species from the adsorbent structure after the adsorption process was also evaluated. The residual potassium concentration in the treated solution was quantified by atomic absorption spectroscopy (AAS).

## Results and discussion

### Material characteristics

**Fig. 1:**
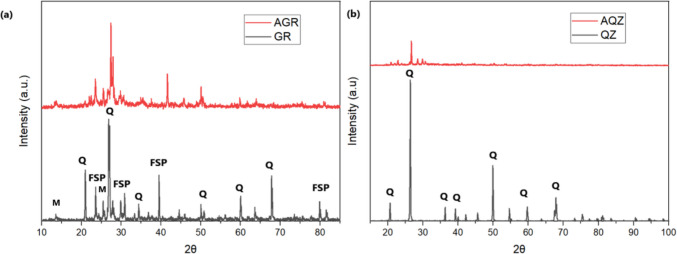
XRD patterns for (**a**) GR and AGR, and (**b**) QZ and AQZ samples. (Q: Quartz, FSP: Feldspar, M: Mica)

The X-ray diffraction pattern of the raw granite sample (Fig. 1a) indicates a highly crystalline and polymineralic material, as evidenced by the presence of numerous sharp and well-defined diffraction peaks (Bakruddin and Sembiring 2019). The diffractogram shows prominent reflections attributed to α-quartz (Q), with the most intense peak observed at approximately 2θ ≈ 26.6°, corresponding to the (101) crystallographic plane (Hillier 1994). Additional quartz-related reflections are observed at around 20.8°, 36.5°, 50.1°, and 59.9°, and in the 68–70° range, confirming the presence of well-crystallized quartz domains (Hillier 1994). Besides quartz, several diffraction peaks located mainly between 22–30° and at higher angles (≈40–80°) are attributed to feldspar phases (FSP), including potassium feldspar (orthoclase or microcline) and sodic plagioclase (albite), which are typical constituents of granitic rocks (Ward et al. 1999). XRD peaks associated with mica are observed at 8.9° and 26.8° (Senthilnathan et al. 2019). Minor and less intense reflections observed throughout the diffractogram may also be associated with micaceous minerals (Ward et al. 1999). The wide angular distribution, sharpness, and high intensity of the diffraction peaks reflect a high degree of crystallinity and long-range structural order, consistent with the mineralogical composition of untreated granitic materials (Bakruddin and Sembiring 2019). The XRD pattern of the raw quartzite sample (Fig. 1b) is dominated by reflections attributed to α-quartz (SiO₂), indicating that quartz is the predominant mineralogical phase (Hillier 1994). The most intense and characteristic quartz reflection is observed at approximately 2θ ≈ 26.6°, accompanied by secondary quartz peaks at around 20.8°, 36.5°, 39.5°, 50.1°, 59.9°, and 68–70°, which are consistent with standard diffraction data for crystalline quartz (Hillier 1994). The sharpness and high intensity of these reflections indicate a high degree of crystallinity and structural order, typical of quartz-rich metamorphic rocks (Kukartsev et al. 2022). Only minor, low-intensity reflections are detected, suggesting the presence of trace accessory phases, mainly feldspars and possibly minor micaceous minerals, occurring in subordinate amounts relative to quartz (Ward et al. 1999).

In both diffraction patterns of the alkali-fused samples (red lines, AGR and AQZ), a pronounced reduction in the intensity of the crystalline diffraction peaks is observed as a direct consequence of the amorphization process induced by alkaline fusion. This treatment promotes the progressive breakdown of the Si–O–Si network and the disruption of long-range crystalline order, leading to a marked attenuation or complete disappearance of the sharp reflections originally associated with quartz and aluminosilicate phases. The appearance of a broad diffuse background further confirms the formation of predominantly amorphous structures. These results demonstrate that solid-state alkaline fusion is an effective method for inducing structural disorder and promoting amorphization of both granitic and quartzitic mineral matrices. These changes are expected to play a decisive role in improving the functional performance of AGR and AQZ, especially in adsorption applications.

Table 1 summarizes the elemental composition obtained by XRF analysis for the raw rocks (GR and QZ) and their alkali-treated counterparts (AGR and AQZ). The granite precursor (GR) is dominated by silicon and aluminum, reflecting its typical aluminosilicate mineralogy (El-Taher 2012). After treatment with alkaline fusion, the AGR sample shows an increase in potassium content, accompanied by a decrease in the relative proportion of Si. This shift indicates that the alkaline fusion process not only introduces potassium into the material but also promotes the breakdown of the original silicate network, resulting in structural reorganization and the formation of amorphous phases (Shoppert et al. 2019). An analogous behavior is observed for the quartzite system. The QZ sample shows an extremely high Si content (91.22 wt.%), consistent with its predominantly quartz composition (Finestone et al. 2020). Following alkaline fusion, the AQZ material shows a decrease in Si, accompanied by an increase in K, confirming the effective interaction between KOH and the quartz structure (Shoppert et al. 2019). This compositional modification suggests that even highly crystalline silica phases can be significantly altered by alkaline fusion, leading to a partial or total amorphization of the crystalline structure. (Shoppert et al. 2019).

**Table 1:** Chemical composition (wt.%) of GR, AGR, QZ, and AQZ samples

Element	GR (%)	AGR (%)	QZ (%)	AQZ (%)
Si	62.817	49.322	91.216	61.822
K	14.896	26.832	1.311	34.251
Al	11.049	14.299	1.586	1.212
Fe	5.651	4.288	0.092	0.22
Ca	2.379	2.707	1.528	1.546
P	1.79	1.406	2.933	0.614
S	0.808	0.662	1.291	0.295
Ti	0.446	0.31	-	-
Mn	0.078	0.06	-	-
Rb	0.057	0.048	-	-
Zn	0.015	0.019	0.006	0.001
Sr	0.007	0.008	-	-
Cu	0.007	0.039	0.037	0.039

**Fig. 2:**
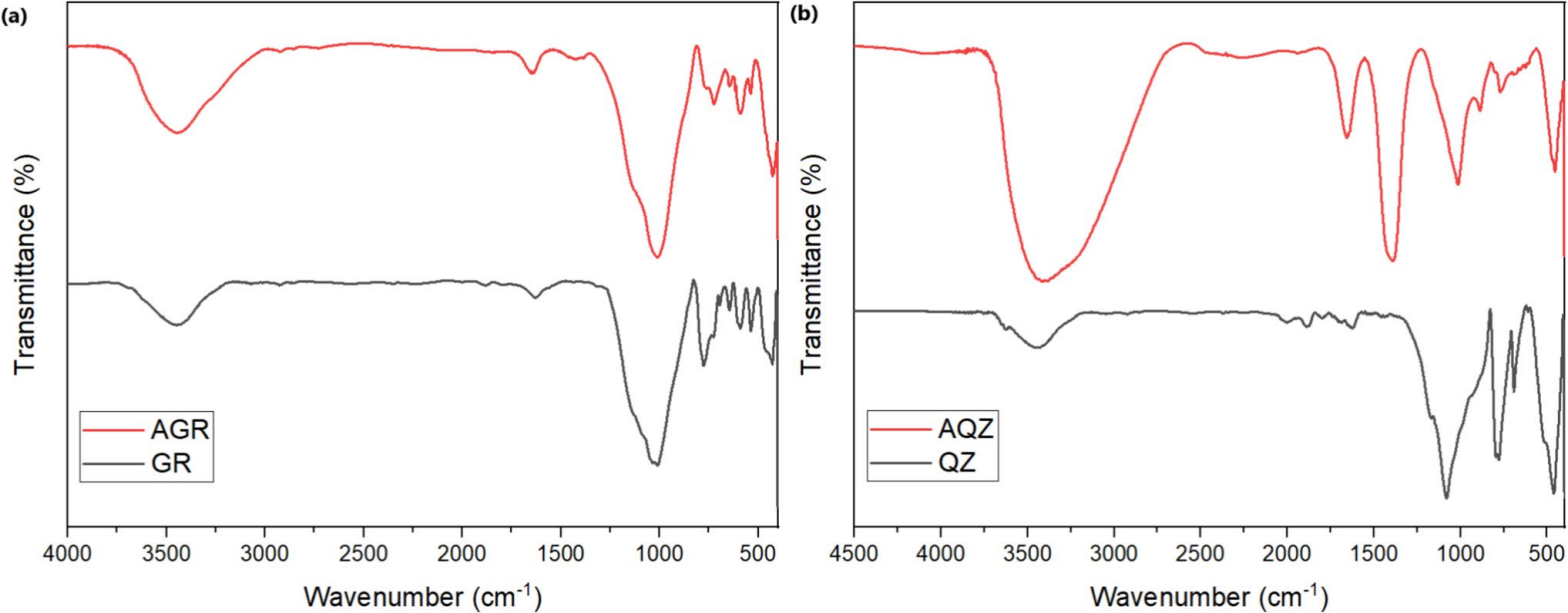
FTIR patterns for (**a**) GR and AGR, and (**b**) QZ and AQZ samples

Fourier transform infrared (FTIR) spectra of granite (GR) and quartzite (QZ), before and after alkaline treatment, are presented in Fig. 2a and b, respectively. The gray curves show the spectra of the untreated materials, while the red curves represent the treated samples (AGR and AQZ). Analysis of these spectra provides insight into the materials’ structural composition and the chemical modifications induced by the surface treatment.

FTIR spectrum of untreated granite (GR) exhibits features characteristic of a complex silicate-based material, consistent with its mineralogical composition, which commonly includes quartz, feldspars, and minor accessory phases (El-Taher 2012). The most prominent absorption band appears in the 1000–1100 cm⁻^1^ region and is attributed to the asymmetric stretching vibrations of Si–O–Si bonds within the silicate framework (Coulibaly et al. 2016). This band confirms the predominance of silica-containing minerals in the granite structure. Additional absorptions near 780–800 cm⁻^1^ are associated with symmetric stretching modes of Si–O bonds, particularly related to quartz, while bands observed around 450–500 cm⁻^1^ correspond to bending vibrations of Si–O linkages (Coulibaly et al. 2016; Li et al. 2014). In the higher wavenumber region, a broad band centered around approximately 3400 cm⁻^1^ is assigned to O–H stretching vibrations, and a weaker band near 1630 cm⁻^1^ is related to the bending mode of molecular water (H–O–H). The relatively low intensity of these hydroxyl-related bands indicates a limited amount of surface hydroxyl groups and physically adsorbed water in the untreated granite. After surface treatment, noticeable spectral changes are observed in the AGR sample. In particular, there is a clear increase in the intensity of the broad band in the 3200–3600 cm⁻^1^ region, indicating enhanced surface hydroxylation (Coulibaly et al. 2016). This increase can be attributed to the formation of silanol (Si–OH) groups resulting from the partial hydrolysis of siloxane (Si–O–Si) bonds at the mineral surface (Paul et al. 2007). Minor variations in the shape and intensity of the main Si–O–Si stretching band further suggest modifications in the surface structure of the silicate network (Coulibaly et al. 2016; Li et al. 2014). These changes confirm that the treatment successfully altered the granite surface, promoting the formation of active hydroxylated sites (Coulibaly et al. 2016; Li et al. 2014).

The FTIR spectra of QZ (gray curve) and AQZ (red curve) clearly demonstrate the structural modifications induced by the alkaline activation process. The QZ sample exhibits spectral features characteristic of highly crystalline quartz (Coulibaly et al. 2016). The spectrum is dominated by a sharp, strong band centered at 1080–1100 cm⁻^1^, assigned to the asymmetric stretching vibrations of Si–O–Si bonds, which constitute the main structural framework of α-quartz. Additional well-defined bands at approximately 800 and 780 cm⁻^1^ correspond to symmetric stretching modes, while the band near 460–470 cm⁻^1^ is attributed to Si–O–Si bending vibrations (Coulibaly et al. 2016; Li et al. 2014). The narrowness and high intensity of these peaks indicate a high degree of structural order and crystallinity (Shoppert et al. 2019). In the high-wavenumber region, only weak bands around 3400 cm⁻^1^ (O–H stretching) and ~ 1630 cm⁻^1^ (H–O–H bending) are observed, suggesting limited surface hydroxylation and minor amounts of physically adsorbed water.

In contrast, the AQZ spectrum reveals significant band broadening and reduced peak definition, particularly in the 1200–800 cm⁻^1^ region, indicating partial disruption of the crystalline quartz framework. The main Si–O–Si asymmetric stretching band broadens and shifts slightly, reflecting decreased long-range order and the formation of an amorphous silicate phase (Coulibaly et al. 2016). The bands at ~ 800 and ~ 780 cm⁻^1^ become less sharp, further confirming the loss of crystallinity. Additionally, the broad and intensified band around 3400 cm⁻^1^ and the more evident feature near 1630 cm⁻^1^ indicate an increased concentration of surface hydroxyl groups and adsorbed water, likely resulting from the generation of silanol (Si–OH) groups during alkaline fusion. The widening and enhancement of bands in the 950–1000 cm⁻^1^ region suggest the formation of new reactive silicate species, possibly associated with Si–O⁻ groups or modified silicate environments (Ellerbrock et al. 2022). Overall, while QZ retains a well-ordered crystalline silica structure, AQZ displays clear evidence of structural reorganization, partial amorphization, and increased surface hydroxylation (Ito and Nakashima 2002). These modifications are consistent with the development of a more reactive and potentially more adsorptive material after alkaline fusion (Paul et al. 2007).

**Fig. 3:**
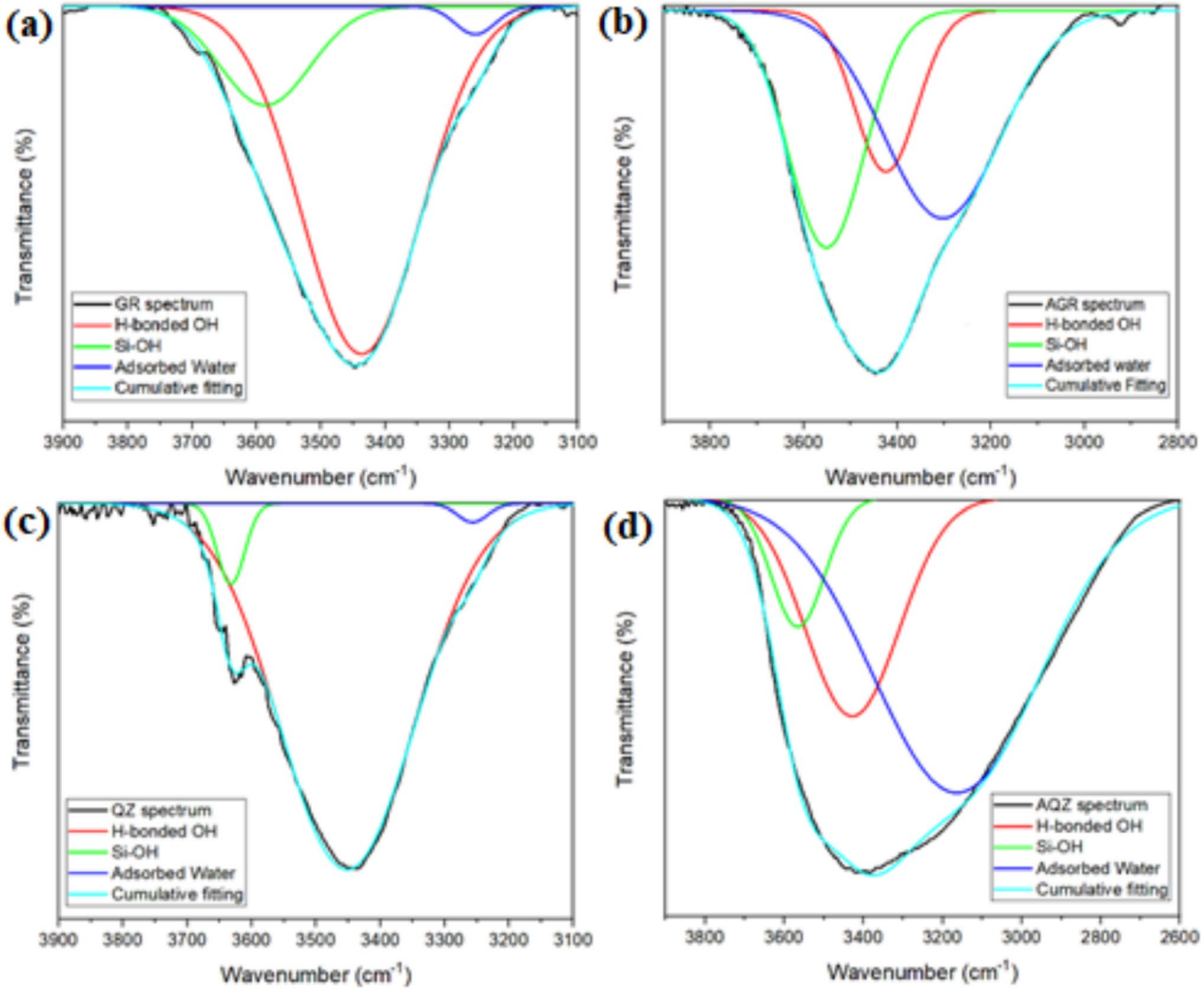
FTIR deconvolution of the 3200–3800 cm^−1^ region for GR (**a**), AGR (**b**), QZ (**c**), and AQZ (**d**) samples

Also, Fig. 3 presents the deconvolution of the FTIR spectra in the 3200–3800 cm^−1^ region for the GR, AGR, QZ, and AQZ samples. This spectral region is commonly associated with O–H stretching vibrations and was resolved into three main contributions attributed to H-bonded hydroxyl groups, isolated Si–OH groups, and adsorbed water molecules (Coulibaly et al. 2016; Paul et al. 2007). The deconvolution procedure enabled the quantitative determination of the relative contribution of each component peak to the total band area, as summarized in Table 2. The GR sample exhibited a predominance of H-bonded hydroxyl groups, corresponding to 80.37% of the total area, while the contributions of Si–OH groups and adsorbed water were comparatively low, accounting for 17.41% and 2.22%, respectively. A similar behavior was observed for the QZ sample, which presented an even higher proportion of H-bonded OH species (94.71%), indicating a surface dominated by strongly interacting hydroxyl groups and limited water adsorption capacity.

**Table 2:** Relative area (%) of deconvoluted O–H bands for GR, AGR, QZ, and AQZ samples

Sample	H-bonded OH	Si–OH	Adsorbed water
GR	80.37	17.41	2.22
AGR	19.10	34.39	46.51
QZ	94.71	4.22	1.07
AQZ	26.73	8.98	64.29

After chemical activation with KOH, significant modifications in the hydroxyl environment were observed for both materials. The AGR sample showed a marked reduction in the H-bonded OH contribution from 80.37 to 19.10%, accompanied by a substantial increase in the adsorbed water fraction to 46.51%. Likewise, the AQZ sample exhibited a decrease in H-bonded OH groups from 94.71 to 26.73%, whereas the adsorbed water contribution increased dramatically to 64.29%. These results indicate that KOH activation strongly altered the surface chemistry of the materials by disrupting hydrogen-bonded hydroxyl networks and generating a more hydrophilic surface with enhanced affinity for water molecules (Ito and Nakashima 2002). The increase in adsorbed water content after activation can be associated with the formation of new surface defects, pore development, and exposure of oxygen-containing functional groups during the alkaline treatment (Ellerbrock, Stein, and Schaller 2022). In addition, the partial increase in the Si–OH contribution observed for AGR suggests that KOH treatment promoted the exposure or generation of silanol groups due to structural rearrangements and partial dissolution of mineral phases (Paul et al. 2007).

The deconvolution profiles shown in Fig. 3 further corroborate these findings, as the activated samples exhibited broader and more intense contributions associated with adsorbed water at lower wavenumbers, characteristic of physically adsorbed and weakly coordinated water molecules. Such modifications demonstrate that KOH activation significantly enhanced the surface reactivity and hydrophilicity of the materials, which may directly contribute to improved adsorption performance by increasing the availability of active sites and facilitating interactions with aqueous species (Figueiredo et al. 2024).

Overall, both granite and quartzite present FTIR spectra dominated by vibrations associated with silicate networks, confirming their silica-based nature. However, granite exhibits a more complex spectral profile due to its multiphase mineralogy, whereas quartzite shows sharper, more defined quartz-related features (El-Taher 2012). The observed increase in hydroxyl-related bands after treatment in both materials demonstrates the effectiveness of the surface modification process in generating hydroxylated functional groups. The higher degree of surface hydroxylation observed for quartzite suggests a greater density of active sites, which may positively influence its performance in applications where surface interactions, such as adsorption processes, are crucial (Qiu et al. 2024; Zhou et al. 2026).

**Fig. 4:**
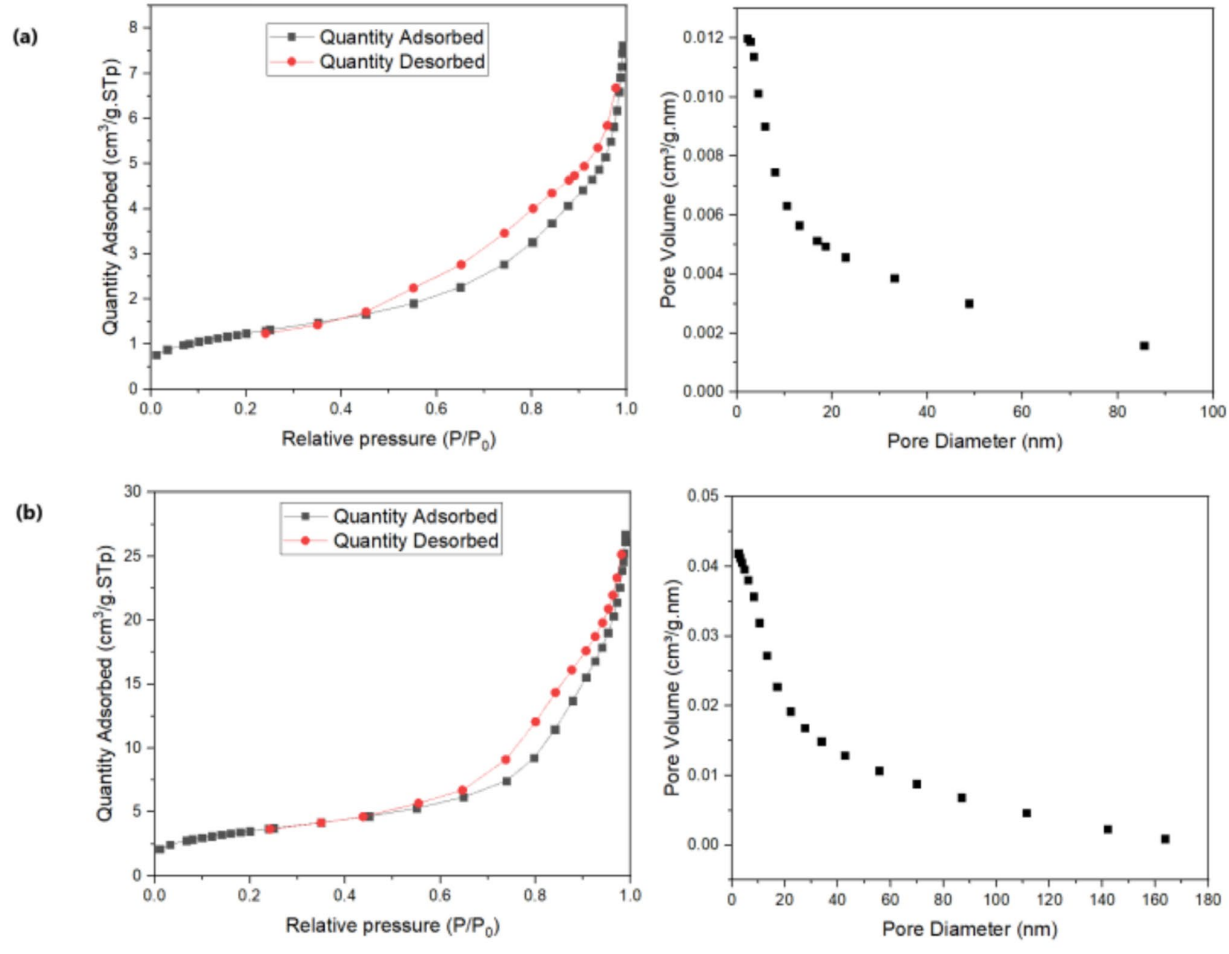
Nitrogen adsorption–desorption isotherms and pore size distribution curves for (**a**) AGR and (**b**) AQZ samples

The textural properties of the raw and amorphized samples were evaluated using N₂ adsorption–desorption isotherms and pore size distribution analyses (Fig. 4). All samples exhibited type IV isotherms, characteristic of mesoporous materials, confirming that the pore structure is predominantly composed of mesopores (2–50 nm range). This interpretation is further supported by the pore size distribution profiles, which indicate that the pore diameters of all materials fall within the mesoporous region, in agreement with (Thommes et al. 2015). The quantitative textural parameters (Table 3) demonstrate a pronounced effect of alkaline fusion on both granite- and quartzite-derived materials. The untreated granite sample (GR) displayed extremely low surface area (0.24 m^2^ g⁻^1^) and pore volume (0.0005 cm^3^ g⁻^1^), indicating a nearly non-porous structure. After alkaline fusion, the amorphized granite (AGR) exhibited a substantial increase in surface area (6.23 m^2^ g⁻^1^) and pore volume (0.011 cm^3^ g⁻^1^), along with a slight reduction in average pore diameter (from 10.93 to 9.54 nm). These changes suggest the generation of new mesopores and the development of a more accessible porous network.

**Table 3:** Pore properties for GR, AGR, QZ, and AQZ samples

Sample	Surface area (m^2^ g^−1^)	Pore volume (cm^3^ g^−1^)	Average pore size (nm)
GR	0.24	0.0005	10.93
AGR	6.23	0.011	9.54
QZ	0.33	0.0006	6.54
AQZ	17.59	0.041	12.27

A similar trend is observed for the quartzite-derived materials. The raw quartzite (QZ) showed low surface area (0.33 m^2^ g⁻^1^) and pore volume (0.0006 cm^3^ g⁻^1^), while the amorphized quartzite (AQZ) exhibited a marked enhancement, reaching 17.59 m^2^ g⁻^1^ and 0.041 cm^3^ g⁻^1^, respectively. In contrast to AGR, AQZ also showed an increase in average pore size (from 6.54 to 12.27 nm), indicating not only pore formation but also pore widening and structural rearrangement during the amorphization process. Overall, the results clearly demonstrate that alkaline fusion effectively transforms both granite and quartzite into highly porous amorphous materials. The most significant improvement is observed for AQZ, which presents the highest surface area and pore volume among all samples, indicating the formation of a more disordered and accessible porous framework. This enhanced mesoporosity is particularly favorable for adsorption applications and other surface-dependent processes (Hosseinzadeh et al. 2015).

### Comparison of adsorption efficiency for different dyes and materials

Based on the results presented in Table 4, the amorphized materials AGR and AQZ demonstrated markedly superior adsorption performance compared with their respective precursor materials, GR and QZ. This improvement can be attributed to the alkaline fusion process, which induces significant structural modifications, including the formation of new surface functional groups, an increase in specific surface area, and enhanced pore development. These changes increase the number of accessible active sites, thereby improving the adsorption capacity of the treated materials. For the GR–AGR system, AGR consistently exhibited higher adsorption capacities (*q*) and removal efficiencies (*R*) for all evaluated dyes. For Congo Red (CR), q increased from 1.83 to 5.27 mg g⁻^1^, with the corresponding removal efficiency rising from 3.66 to 10.54%. For Methyl Orange (MO), the adsorption capacity increased from 2.17 to 6.72 mg g⁻^1^, while removal efficiency improved from 4.34 to 13.44%. A more pronounced enhancement was observed for the cationic dyes. Methylene Blue (MB) showed an increase in q from 4.31 to 19.33 mg g⁻^1^ and in R from 8.62 to 38.66%, whereas Crystal Violet (CV) increased from 5.28 to 22.43 mg g⁻^1^, with removal efficiency rising from 10.56 to 44.86%. These results clearly indicate that AGR has a significantly greater affinity for both anionic and cationic dyes than GR.

**Table 4:** Adsorption efficiency of Congo Red (CR), Methyl Orange (MO), Methylene Blue (MB), and Crystal Violet (CV) onto GR, AGR, QZ, and AQZ samples

Sample	GR		AGR		QZ		AQZ	
Dye	*q* (mg g^−1^)	*R* (%)	*q* (mg g^−1^)	*R* (%)	*q* (mg g^−1^)	*R* (%)	*q* (mg g^−1^)	*R* (%)
CR	1.83	3.66	5.27	10.54	2.41	4.82	6.93	13.86
MO	2.17	4.34	6.72	13.44	2.63	5.26	9.78	19.56
MB	4.31	8.62	19.33	38.66	6.79	13.58	30.57	61.14
CV	5.28	10.56	22.43	44.86	7.91	15.82	36.06	72.11

An even more substantial improvement was observed for the QZ–AQZ system. AQZ presented higher adsorption capacities than QZ for all dyes studied. For CR, *q* increased from 2.41 to 6.93 mg g⁻^1^, and the removal efficiency rose from 4.82 to 13.86%. For MO, *q* increased from 2.63 to 9.78 mg g⁻^1^ and R from 5.26 to 19.56%. The enhancement was particularly remarkable for MB and CV. In the case of MB, the adsorption capacity increased from 6.79 to 30.57 mg g⁻^1^, accompanied by an increase in removal efficiency from 13.58 to 61.14%. For CV, *q* increased from 7.91 to 36.06 mg g⁻^1^, and the removal efficiency reached 72.11%, representing the highest value among all tested systems. Overall, AQZ exhibited the best adsorption performance, especially toward Crystal Violet, suggesting stronger interactions between the modified surface and the dye’s molecular structure. The superior behavior of AQZ can be attributed to higher porosity and a greater number of active functional groups on the adsorbent surface. Given its outstanding adsorption capacity and removal efficiency, AQZ was selected for further investigation, including optimization of operational parameters (e.g., adsorbent dosage and solution pH) and detailed kinetic, equilibrium, and thermodynamic studies, to comprehensively evaluate its applicability in aqueous effluent treatment systems.

### Optimization of pH and AQZ dosage

The optimal conditions for pH and adsorbent dosage were determined using the CCRD 2^2^ experimental design. Table 5 presents the matrix with both coded and real values.

**Table 5:** Experimental design 2^2^ matrix and responses for the CV adsorption onto the AQZ sample

Run	Adsorbent dosage (g L^−1^)	pH	*q* (mg g^−1^)	*R* (%)
1	0.36 (−1)	4.9 (−1)	41.23 ± 0.21	28.17 ± 0.18
2	0.89 (1)	4.9 (−1)	34.86 ± 0.27	55.34 ± 0.22
3	0.36 (−1)	9.1 (1)	51.83 ± 0.19	48.27 ± 0.25
4	0.89 (1)	9.1 (1)	48.91 ± 0.23	74.93 ± 0.17
5	0.25 (−1.41)	7 (0)	50.67 ± 0.29	30.28 ± 0.21
6	1.0 (1.41)	7 (0)	38.24 ± 0.16	65.19 ± 0.26
7	0.625 (0)	4(1.41)	36.97 ± 0.24	48.14 ± 0.19
8	0.625 (0)	10 (1.41)	49.87 ± 0.18	69.82 ± 0.28
9	0.625 (0)	7 (0)	44.36 ± 0.22	60.41 ± 0.15
10	0.625 (0)	7 (0)	43.79 ± 0.17	62.38 ± 0.23
11	0.625 (0)	7 (0)	44.08 ± 0.26	61.27 ± 0.20

Table 6 presents the regression coefficients, pure error, and corresponding *p*-values obtained for adsorption capacity (*q*) and removal efficiency (*R*). A confidence level of 95% (*p* < 0.05) was adopted to determine the statistical significance of the evaluated terms, allowing the identification of the most influential factors governing the adsorption process. For adsorption capacity, the statistical analysis indicated that the linear effect of pH (*x₂*) was significant, demonstrating that solution pH directly influences the amount of dye retained per unit mass of adsorbent. The positive coefficient for x₂ indicates that increasing pH enhances adsorption capacity within the studied range (Mouelhi et al. 2016). Although the linear effect of adsorbent dosage (*x₁*) did not appear statistically significant in the model, it is well established that adsorption capacity is intrinsically dependent on the amount of adsorbent employed (Mouelhi et al. 2016). Therefore, the lack of statistical significance may be attributed to a masking effect from the strong pH influence, which may have overshadowed the dosage contribution under the evaluated conditions.

In contrast, removal efficiency was significantly affected by both the linear term of adsorbent dosage (*x₁*) and the linear term of pH (*x₂*), indicating that these variables independently contribute to the percentage of dye removal (Imessaoudene et al. 2023). The positive coefficients indicate that increasing either the adsorbent dosage or the solution pH results in higher removal efficiency (Imessaoudene et al. 2023). Based on the parameters identified as statistically significant at the 95% confidence level, predictive mathematical models were developed, yielding Eqs. 12 and 13. These equations provide an adequate representation of the system and can be reliably applied to estimate the adsorption capacity and removal efficiency within the investigated experimental range.

12 $$q=32.11+2.42x2$$

13 $$R=40.14+29.92x1+3.38x2$$

where × *1* is the adsorbent dosage and × *2* is the pH in coded values.

**Table 6.** *p*-values, pure error, and estimated effects of response factors on the 2^2^ experimental design

Factor	*R* (%)			*q* (mg g^−1^)		
	Effect	Pure error	*p*	Effect	Pure error	*p*
Mean	40.14	2.75	0.000028	32.11	1.97	0.000016
AD (L)	29.92	3.38	0.00030	−2.57	2.42	0.33
AD (Q)	−0.91	4.03	0.82	1.15	2.89	0.70
pH (L)	23.83	3.38	0.00089	19.16	2.42	0.00052
pH (Q)	−8.06	4.03	0.10	−7.26	2.89	0.05
AD X pH	8.41	4.77	0.13	−0.43	3.42	0.90

**Fig. 5:**
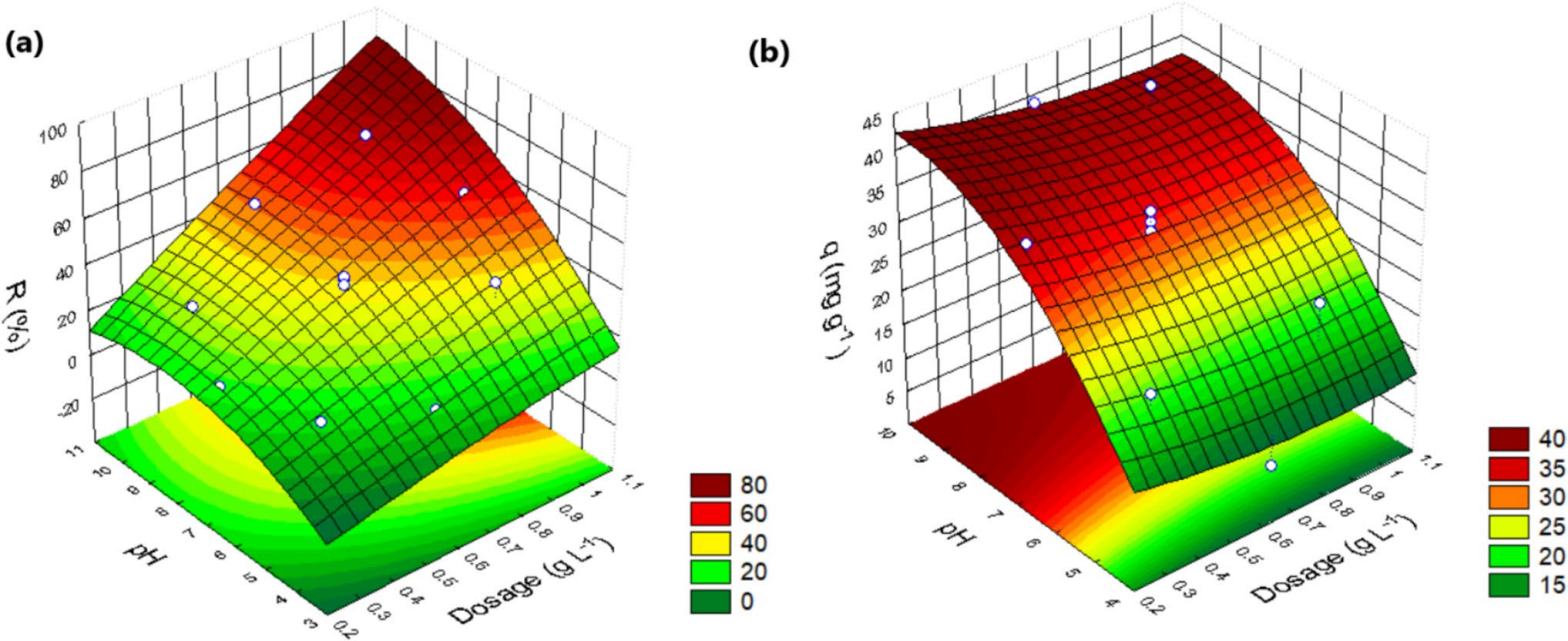
Response surface for (**a**) *q* and (**b**) *R*

The statistical adequacy of the experimental data is essential to ensure that the proposed model accurately represents the system behavior. Table 7 presents the coefficients of determination (*R*^2^) obtained for both response variables. High *R*^2^ values were achieved, namely 0.93 for adsorption capacity and 0.96 for dye removal efficiency, indicating a strong correlation between the experimental and predicted results and confirming the good fit of the models. Additionally, the Fisher test further supports the reliability of the developed models, as the calculated *F*-values exceeded the corresponding tabulated critical values (Mouelhi et al. 2016). This trend demonstrates the regression equations’ statistical significance and predictive capability. Consequently, the validated models were used to construct the response surface plots of adsorption capacity (*q*) and removal efficiency (*R*), as illustrated in Fig. 5.

**Table 7:** Statistical parameters of quadratic models for the responses R (%) and q

Variable	R^2^	F_calc_	F_standard_
*q* (mg g^−1^)	0.93	14.24	2.98
*R* (%)	0.96	26.99	2.98

Figure 5 indicates that the highest adsorption capacity is achieved under alkaline conditions. This trend is associated with the cationic character of crystal violet (CV), which promotes strong electrostatic interactions with negatively charged surfaces. Since the AQZ exhibits a point of zero charge (pHₚzc) of 6.7, the adsorbent surface becomes predominantly negatively charged at pH values above this point. As a result, electrostatic attraction between negatively charged surface sites and positively charged CV molecules is strengthened, leading to enhanced adsorption performance. Conversely, at acidic pH, surface protonation occurs, decreasing the density of negatively charged active sites and consequently reducing adsorption capacity. Based on these observations, the most favorable operational parameters for CV removal by AQZ were determined to be pH 9 and an adsorbent dosage of 0.9 g L⁻^1^. These optimized conditions were therefore employed in the subsequent kinetic and equilibrium investigations, as they promote stronger dye–adsorbent interactions and provide more consistent and reliable modeling outcomes.

### Kinetics results

The kinetic parameters presented in Table 8 indicate that all evaluated models adequately describe the adsorption of CV onto the AQZ sample; however, noticeable differences in fitting quality are observed among them. For the pseudo-first-order model, the calculated adsorption capacities (*q₁*) increased with the initial dye concentration, ranging from 48.53 to 78.57 mg g⁻^1^ for C₀ values between 50 and 350 mg L⁻^1^. The rate constant (*k₁*) did not show a strictly proportional relationship with concentration, and the average relative error (*ARE*) remained high (13.61–18.40%), despite *R*^*2*^ values between 0.95 and 0.98. These results suggest that, although the model provides a reasonable correlation with the experimental data, it does not fully represent the adsorption kinetics over the entire concentration range.

**Table 8:** Kinetics models data for CV adsorption onto AQZ sample

Pseudo-first-order				
C_0_ (mg L^−1^)	50	150	250	350
*q*_*1*_ (mg g^−1^)	48.53	60.06	69.00	78.57
*k*_*1*_ (min^−1^)	0.04	0.05	0.008	0.013
*ARE* (%)	18.40	14.04	15.83	13.61
*R*^*2*^	0.98	0.98	0.96	0.95
Pseudo-second Order				
*q*_*2*_ (mg g^−1^)	56.58	68.48	76.51	85.84
*k*_*2*_ (g mg^−1^ min^−1^)	0.0009	0.001	0.0013	0.0017
*ARE* (%)	12.16	9.14	7.77	5.01
*R*^*2*^	0.98	0.98	0.98	0.99
Avrami				
*q*_*AV*_ (mg g^−1^)	50.77	61.91	73.38	85.14
*k*_*AV*_ (min^−1^)	0.078	0.09	0.16	0.24
*n* (-)	0.78	0.79	0.64	0.56
*ARE* (%)	7.96	6.76	3.77	1.55
*R*^*2*^	0.99	0.99	0.99	0.99

The pseudo-second-order model showed improved predictive capability. The estimated equilibrium adsorption capacities (*q₂*) increased from 56.58 to 85.84 mg g⁻^1^ as the initial concentration rose. The coefficients of determination were consistently high (*R*^*2*^ = 0.98–0.99), and the *ARE* values were significantly lower than those of the pseudo-first-order model, reaching a minimum of 5.01% at 350 mg L⁻^1^. This behavior indicates a more accurate representation of the adsorption mechanism, possibly involving stronger interactions between the CV molecules and the AQZ surface.

**Fig. 6:**
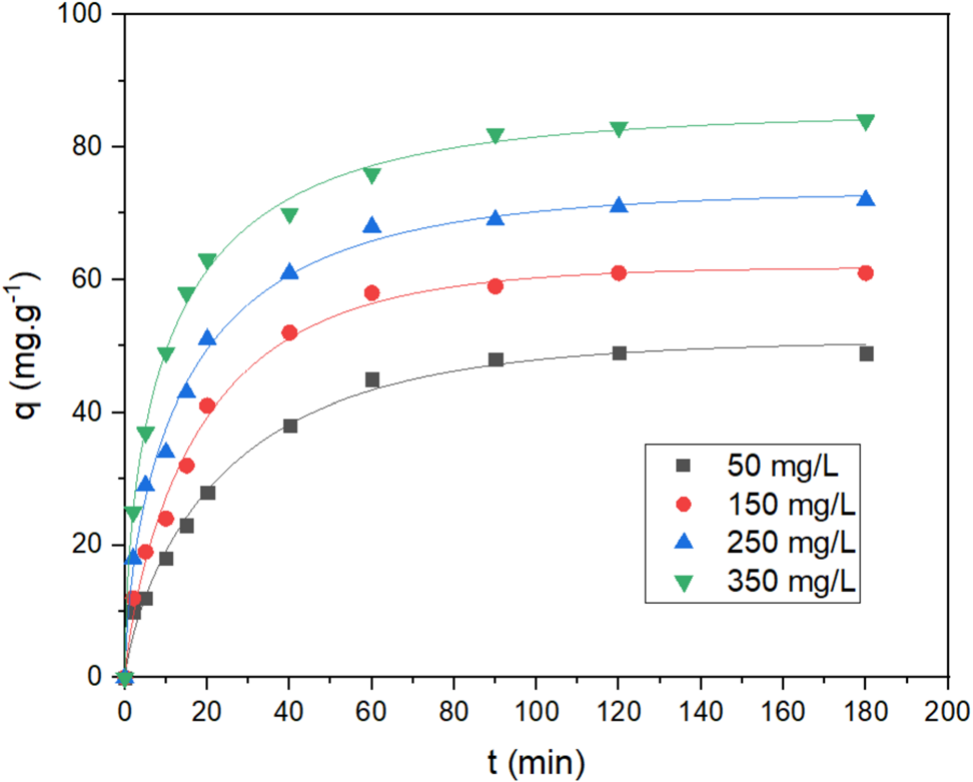
Adjustment of the kinetic model of Avrami

Among the evaluated models, the Avrami equation provided the best overall fit. The calculated capacities (*q*_*AV*_) closely followed the experimental trend, varying from 50.77 to 85.14 mg g⁻^1^ with increasing initial concentration. The rate constant (*k*_*AV*_) increased progressively from 0.078 to 0.24 min⁻^1^, suggesting enhanced adsorption rates at higher dye concentrations. Notably, this model exhibited the lowest *ARE* values (1.55–7.96%) and consistently high determination coefficients (*R*^*2*^ = 0.99 for all concentrations), indicating excellent agreement with the experimental results. The Avrami exponent (*n*) ranged from 0.56 to 0.79, with values approaching 0.5 at higher concentrations. This pattern suggests that the adsorption process is predominantly diffusion-controlled rather than governed by ideal surface reaction kinetics. The gradual decrease in n with increasing concentration supports the assumption that mass transfer limitations and progressive occupation of active sites play a significant role as equilibrium is approached (Dotto et al. 2012). Thus, from Fig. 6, it is evident that AQZ required 90 min to reach saturation in the kinetic assay. Overall, based on the statistical indicators and the physical interpretation of the kinetic constants, the Avrami model most accurately describes the adsorption kinetics of CV onto AQZ, suggesting a complex, multi-step mechanism influenced by diffusion.

### Equilibrium results

Table 9 presents the isotherm parameters obtained from fitting the equilibrium data of CV adsorption onto the AQZ sample at different temperatures (298–328 K). Among the evaluated models, the Freundlich equation provided the best fit to the experimental results. This statement is supported by high coefficients of determination (*R*^2^ = 0.97–0.99) and relatively low average relative errors (ARE = 3.90–8.16%), particularly at 308–328 K, confirming strong agreement between the calculated and experimental data. These findings suggest that the adsorption process occurs on a heterogeneous surface with energetically non-uniform active sites. The Freundlich constant ${K}_{F}$, which is associated with adsorption capacity, decreased from 31.62 to 10.09 as temperature increased, indicating a reduction in adsorption affinity at higher temperatures. Similarly, the Langmuir maximum adsorption capacity (${Q}_{max}$) showed a slight decline from 74.94 mg g⁻^1^ at 298 K to 68.93 mg g⁻^1^ at 328 K. Although the Langmuir model presented reasonable fits (*R*^*2*^ = 0.86–0.94), its higher *ARE* values (11.16–13.47%) compared to the Freundlich model indicate a less accurate representation of the equilibrium data, suggesting that monolayer adsorption on a homogeneous surface is not the predominant mechanism.

**Table 9:** Isotherms models data for CV adsorption onto the AQZ sample

Freundlich				
T (K)	298	308	318	328
*K*_*F*_ (mg g^−1^) (mg L^−1^)^−1/n^_F_	31.62	21.66	14.98	10.09
*n*_*F*_ (-)	6.10	4.56	3.80	3.20
*ARE* (%)	8.16	3.90	4.45	4.80
*R*^*2*^	0.97	0.99	0.99	0.99
Langmuir				
*K*_*L*_ (L mg^−1^)	0.23	0.06	0.03	0.02
${Q}_{max}$(mg g^−1^)	74.94	76.51	72.10	68.93
*ARE* (%)	13.47	12.05	12.19	11.16
*R*^*2*^	0.86	0.93	0.93	0.94
Temkin				
*K*_*T*_ (L mg^−1^)	18.01	1.65	0.64	0.29
${\beta}_{T}$(J mol^−1^)	9.17	11.99	12.57	13.14
*ARE* (%)	9.65	6.33	7.32	7.52
*R*^*2*^	0.95	0.98	0.98	0.97

**Fig. 7:**
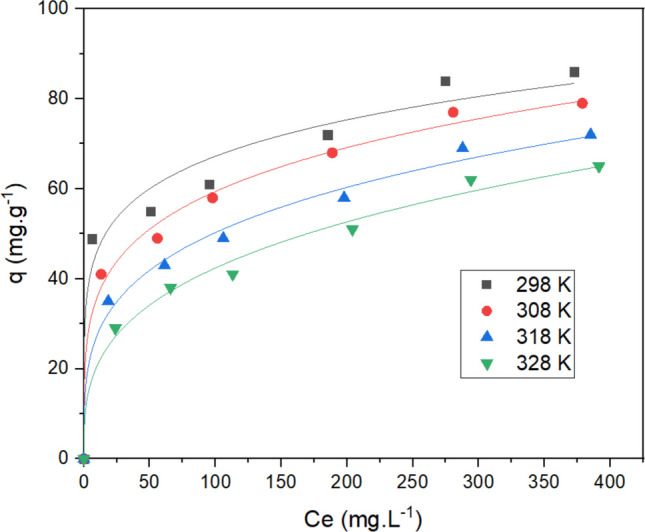
Adjustment of the isotherm model of the Freundlich

The Temkin model also showed satisfactory correlation (*R*^*2*^ = 0.95–0.98) with moderate *ARE* values (6.33–9.65%). The increase in the Temkin constant ${\beta}_{T}$ with temperature indicates variations in the heat of adsorption, reflecting interactions between the adsorbate and the adsorbent at varying surface coverages (Aljeboree et al. 2015). As illustrated in Fig. 7, the adsorption capacity decreases with increasing temperature, confirming that the process is thermodynamically less favorable at elevated temperatures. This behavior may be attributed to the increased solubility of crystal violet in solution at higher temperatures. Greater dye solubility enhances its interaction with water molecules, thereby reducing its affinity for the AQZ surface. Overall, the equilibrium data indicate that the Freundlich model best describes CV adsorption onto AQZ with a maximum adsorption capacity of 86.24 mg.g^−1^, reinforcing the heterogeneous nature of the adsorption surface.

### Adsorption thermodynamics and mechanism

Table 10 shows the data obtained from the adsorption thermodynamics of CV onto the AQZ sample. The thermodynamic parameters presented in Table 10 provide important insights into the nature of CV adsorption onto the AQZ sample. The negative value of *ΔH⁰* (− 0.049 kJ mol⁻^1^) indicates that the process is exothermic. Moreover, the very low magnitude of *ΔH⁰* suggests that the adsorption mechanism is predominantly physical rather than chemical bond formation. The Gibbs free energy values (*ΔG⁰*) are negative at all evaluated temperatures (− 31.07 to − 34.21 kJ mol⁻^1^), confirming that the adsorption process is spontaneous and thermodynamically favorable over the studied temperature range. In addition, the positive *ΔS⁰* value (0.10 kJ mol⁻^1^ K⁻^1^) indicates an increase in disorder at the solid–liquid interface during adsorption, which may be related to molecular rearrangements occurring throughout the process.

**Table 10:** Thermodynamic results for CV adsorption onto the AQZ sample

*T* (K)	ΔH^0^ (kJ mol^−1^)	*ΔS*^*0*^ (kJ mol^−1^ K^−1^)	*ΔG*^*0*^ (kJ mol^−1^)
298	−0.049	0.10	−31.07
308			−32.12
318			−33.16
328			−34.21

Based on these thermodynamic findings, a plausible adsorption mechanism can be proposed. The alkaline treatment applied to the material favors the formation of silanol (Si–OH) groups on the AQZ surface, increasing the number of oxygen-containing functional groups. Considering that the pHₚzc of the material is 6.7, at pH 9, the surface becomes predominantly negatively charged due to the deprotonation of silanol groups to Si–O⁻ species. This negatively charged surface enhances electrostatic attraction with the positively charged amine groups (R₁R₂R₃–N⁺) in the crystal violet molecular structure, which remain protonated under alkaline conditions. In addition to electrostatic interactions, structural changes promoted by the alkaline process, such as partial silica dissolution, increased amorphization, and enhanced surface reactivity, contribute to the greater availability of active adsorption sites. The low enthalpy change further supports that the adsorption mechanism is mainly controlled by physical forces, particularly electrostatic attractions, without the formation of strong covalent bonds. Furthermore, the positive *ΔS⁰* value suggests increased randomness at the interface, likely due to the displacement of water molecules and the reorganization of CV molecules on the AQZ surface during adsorption. Overall, the combined effects of surface charge development, structural modifications induced by alkaline treatment, and favorable thermodynamic parameters explain the high adsorption efficiency of the AQZ sample toward CV under alkaline conditions.

### Regeneration and reuse assays

Table 11 presents the regeneration and reuse results of the AQZ sample over four consecutive adsorption–desorption cycles. The material maintained stable performance during the reuse experiments, although a gradual decline in adsorption efficiency was observed with each cycle. The adsorption capacity (*q*) decreased progressively from 48.91 mg g⁻^1^ in the first cycle to 40.37 mg g⁻^1^ in the fourth cycle. Likewise, the removal efficiency (*R*) decreased slightly, from 74.93 to 68.67% after repeated use. Even with this moderate decrease, AQZ preserved a considerable fraction of its initial adsorption capability, indicating good reusability and structural stability.

**Table 11:** Regeneration and reuse results

Cycle	1	2	3	4
*q* (mg g^−1^)	48.91	45.21	42.38	40.37
*R* (%)	74.93	72.45	71.08	68.67

The residual potassium concentration in the solution after the adsorption experiments was quantified by atomic absorption spectroscopy in order to evaluate the possible leaching of alkali species from the adsorbent structure. The obtained concentration was 8.54 mg L⁻^1^, indicating low potassium release from AQZ, even under alkaline conditions (pH 9). According to literature reports, this concentration range is considered relatively low and does not represent significant environmental concern for aqueous systems (Arienzo et al. 2009). Therefore, the results suggest good chemical stability of AQZ and reinforce its potential applicability as an environmentally safe adsorbent for wastewater treatment processes.

**Fig. 8:**
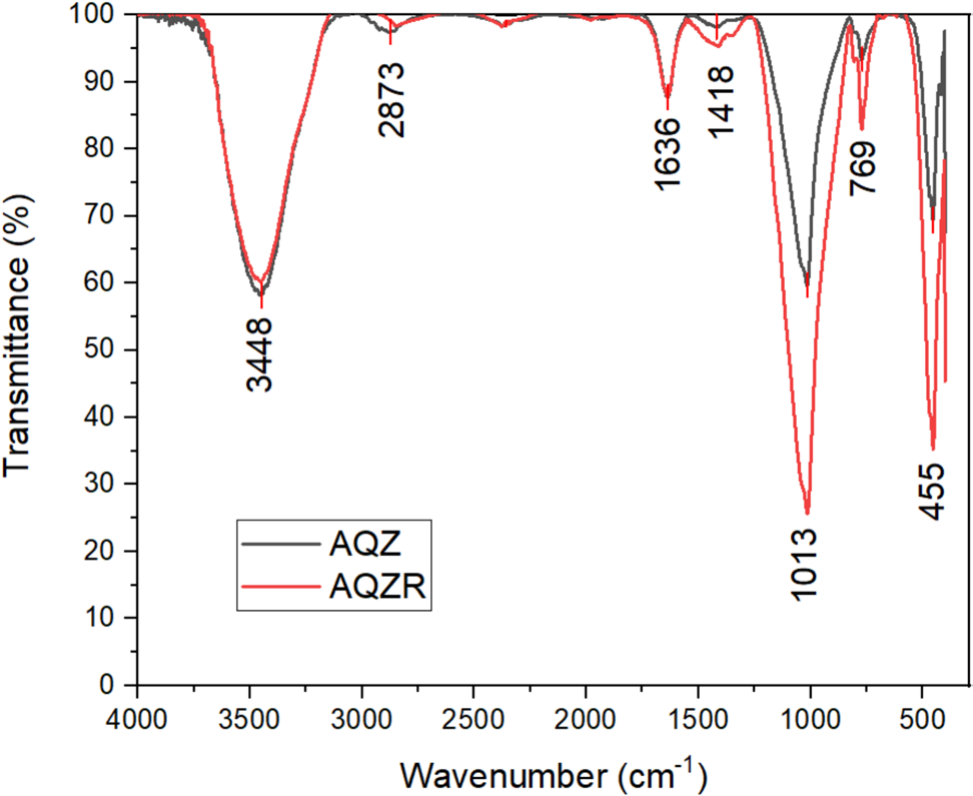
FTIR spectra of AQZ before adsorption and regenerated AQZR after crystal violet adsorption–desorption cycles

The FTIR spectra of AQZ before adsorption and regenerated AQZR after crystal violet (CV) adsorption–desorption cycles are presented in Fig. 8. Both materials exhibited similar spectral profiles, indicating that the alkaline fusion process generated a chemically stable adsorbent structure that was largely preserved after the regeneration procedure. A broad absorption band centered at approximately 3448 cm⁻^1^ was observed for both samples and is attributed to the stretching vibration of hydroxyl groups (O–H) associated with surface silanol groups and adsorbed water molecules. The persistence of this band after regeneration suggests that hydroxyl functionalities remained available on the adsorbent surface, which is important for maintaining adsorption activity. The weak band around 2873 cm⁻^1^ may be associated with C–H stretching vibrations, possibly related to residual organic species or traces of adsorbed dye molecules remaining after regeneration. In addition, the absorption band located near 1636 cm⁻^1^ is assigned to the bending vibration of molecular water (H–O–H), confirming the hydrophilic nature of the adsorbent surface. The band observed at approximately 1418 cm⁻^1^ can be attributed to surface carbonate species or C–N vibrations associated with residual crystal violet molecules. The slight increase in intensity in the regenerated sample suggests that small amounts of dye may remain adsorbed even after the desorption process, which is consistent with the strong interaction between CV molecules and the AQZ surface. The most intense absorption band, located around 1013 cm⁻^1^, corresponds to asymmetric Si–O–Si stretching vibrations, characteristic of silicate-based amorphous structures (Coulibaly et al. 2016). The higher intensity and slight broadening of this band in AQZR may indicate surface interactions between crystal violet molecules and silanol/siloxane groups during adsorption. This result also confirms that the silicate framework remained structurally stable after regeneration. The bands observed near 769 and 455 cm⁻^1^ are attributed to symmetric Si–O vibrations and Si–O bending modes, respectively, which are characteristic of aluminosilicate materials (G. S. Li et al. 2014). The preservation of these bands after regeneration demonstrates that the adsorption–desorption cycles did not significantly alter the structural integrity of the adsorbent. Overall, the FTIR results indicate that AQZ maintained its main functional groups and structural characteristics after regeneration, demonstrating good chemical stability and reusability.

### Comparison of the adsorption capacity for several materials

Table 12 compares the adsorption capacities of various materials reported in the literature for the removal of crystal violet (CV).

**Table 12:** Comparison of the CV adsorption capacity for different adsorbents

Adsorbent	q (mg g⁻^1^)	pH	T (K)	Reference
AQZ sample	86.24	9	298	This work
AGR sample	22.43	9	298	This work
Perlite	1.14	11	303	(Doğan and Alkan 2003)
Carbon jute fiber	27.23	10	303	(Porkodi and Kumar 2007)
Chenopodium album	9.42	8	323	(Arora et al. 2019)
TLAC/chitosan composite	12.5	9	298	(Jayasantha Kumari et al. 2017)
Coniferous Pinus bark	32.78	8	298	(Ahmad 2009)
Palm kernel bark biochar	24.45	10	298	(Kyi et al. 2020)
Peanut shell	12.2	6	298	(Loulidi et al. 2020)
Petiole biochar	24.36	7	303	(Chahinez et al. 2020)
Carbon-iron oxide nanocomposite	58.69	4	298	(Singh et al. 2011)
Polypyrrole-SnO_2_ composite	162.6	7	323	(Kaushik et al. 2026)
Triazine-based organic framework	290	7	298	(Panda et al. 2026)

Among the evaluated adsorbents, the AQZ sample exhibited a remarkable adsorption capacity of 86.24 mg g⁻^1^ at pH 9 and 298 K, outperforming several bio-based and low-cost materials, such as agricultural residues, biochars, and composite materials. Under identical experimental conditions, AQZ showed significantly superior performance compared to the AGR sample, demonstrating its enhanced affinity for CV molecules. Although some chemically modified adsorbents reported higher capacities, these materials generally require more elaborate synthesis or surface treatment processes. In contrast, AQZ offers satisfactory adsorption efficiency, relatively simple preparation, and favorable operational conditions, in addition to good regeneration potential. Therefore, the comparative analysis reinforces the strong applicability of AQZ as an efficient and competitive adsorbent for dye removal systems.

## Conclusions

Ornamental stone residues (granite and quartzite) were successfully transformed into amorphous materials by solid-state alkaline fusion. XRD results confirmed the loss of crystallinity after treatment, and BET analysis indicated a significant enhancement in surface area and pore characteristics. Among the synthesized materials, the amorphous quartzite (AQZ) exhibited the best performance and was therefore chosen for detailed adsorption investigations. The optimum conditions for crystal violet (CV) removal were established at pH 9 with an adsorbent dosage of 0.9 g L⁻^1^. Kinetic evaluation showed that equilibrium was attained within 90 min. Isotherm studies revealed a maximum adsorption capacity of 86.24 mg g⁻^1^ at 298 K, confirming the high efficiency of AQZ for CV uptake. The thermodynamic parameters indicate that the adsorption process is spontaneous, exothermic, and thermodynamically favorable. The mechanism was predominantly associated with electrostatic attraction between negatively charged sites on the AQZ surface and the cationic dye molecules. In addition, AQZ maintained satisfactory performance over four successive adsorption–desorption cycles, indicating good regeneration potential. Overall, this straightforward, efficient strategy demonstrates the feasibility of converting ornamental rock waste into a valuable adsorbent for treating CV-contaminated wastewater. Future investigations will focus on assessing the effects of competing ions, natural organic matter, and synthetic textile effluents on the adsorption performance of AQZ under more complex aqueous environments. In addition, competitive adsorption studies will be carried out to obtain a more detailed understanding of the adsorption mechanism. Moreover, the development of materials with improved porosity and a greater density of surface functional groups may contribute to the production of even more efficient adsorbents for environmental remediation applications.

## Data Availability

The datasets used and analyzed during the current study are available from the corresponding author upon reasonable request.
